# Impact of Antiretroviral Therapy on Intestinal Lymphoid Tissues in HIV Infection

**DOI:** 10.1371/journal.pmed.0030515

**Published:** 2006-12-05

**Authors:** Ronald S Veazey, Andrew A Lackner

## Abstract

Veazey and Lackner discuss a new study which found that most patients who start antiretroviral drugs as early as possible after HIV infection still do not experience complete restoration of intestinal CD4+ T cells to baseline levels.

## Background

Recent studies have confirmed that acute HIV infection results in a rapid and profound loss of memory CD4^+^CCR5^+^ T cells within the first few weeks of infection [1,2]. Since most of these cells reside in the intestinal tract, profound CD4^+^ T cell depletion occurs in the intestine within days of infection, whereas peripheral lymphoid tissues such as blood and lymph nodes, which harbor mainly naïve CD4^+^ T cells, are less severely affected. These studies confirmed earlier reports in macaques infected with simian immunodeficiency virus (SIV) [3] and demonstrated that primary HIV infection results in rapid and dramatic losses of the majority of the CD4^+^ T cells in the body. However, profound losses can only be detected when examining mucosal tissues (i.e., the intestine). Recognition of the mucosal immune system as a principal target of early HIV infection constitutes a fundamental change in our understanding of HIV pathophysiology, with potential implications for therapeutic monitoring and vaccine development [4].

## New Studies in HIV-Infected Patients

Clearly, the massive loss of intestinal memory CD4^+^ T cells imparts profound disturbances in mucosal immunity. However, since mucosal tissues are at best inconvenient to sample, little is known regarding the impact of the loss of this important T cell subset on host immunity or disease progression. In addition, the potential for antiretroviral therapy (ART) to restore mucosal CD4^+^ T cells has been difficult to assess, particularly in acute infection. In a new paper by Mehandru et al. [5], lymphocyte populations from the intestine and peripheral blood were obtained from a relatively large cohort (*n* = 54) of HIV-infected patients soon after infection. These samples were examined and compared with the same samples obtained from HIV-uninfected volunteers to determine whether early therapeutic intervention could restore intestinal CD4^+^ T cells to baseline levels. Prior to this study, few had examined the effects of ART on HIV-infected humans or SIV-infected macaques, particularly inearly infection. A study by Guadalupe et al. had suggested near complete restoration of mucosal CD4^+^ T cells in HIV-infected patients when therapy was initiated early, but only three acutely infected patients were examined [6]. Studies of SIV-infected macaques also suggested that initiating therapy early could result in near complete restoration of mucosal CD4^+^ T cells, but again, few animals were examined [7]. In contrast, Mehandru et al. recently reported a lack of intestinal CD4^+^ T cell repopulation in patients on ART, but this was a cross-sectional study and only eight patients were examined [2]. In summary, although CD4^+^ T cells in the peripheral blood have been reported to fully reconstitute in patients on ART, there is considerable controversy regarding the capacity for restoration of intestinal CD4^+^ T cells, particularly for patients treated in the early stages of infection. This is more than an academic issue, since acutely infected patients are seldom started on ART. Treatment guidelines generally recommend waiting to initiate therapy until blood CD4 cell counts have fallen, in order to reduce the risk of medication side effects as well as the potential for selecting drug-resistant HIV.

The mucosal immune system is a principal target of early HIV infection.

In the current paper, 54 patients in the acute stage of HIV infection were examined and compared with 18 uninfected volunteers. Of the acutely infected patients, 14 were examined only prior to ART, 18 were examined prior to ART and then sequentially through one to three years of uninterrupted therapy, and 22 were examined cross-sectionally at time points ranging from less than one year to seven years after starting ART. Although partial restoration of mucosal CD4^+^ T cells was observed on ART, the results clearly demonstrate that restoration is only partial in the majority of patients compared to uninfected controls, even after several years of “fully suppressive” ART. In addition, these studies demonstrate increased levels of activation in the intestinal immune system of patients on ART, even though immune activation in the peripheral blood often returns to baseline levels. The combined results of this new study suggest that ongoing viral replication and CD4^+^ T cell destruction occur in the intestine of patients on ART, despite what appears to be complete suppression of viral replication in the blood.

## Limitations of HIV Studies

Given the enormous practical challenges involved in obtaining mucosal biopsies from acutely infected patients, the work presented here is certainly valuable. However, a few limitations are inherent in interpreting the results and especially in comparing these data to those from SIV-infected macaques. First, neither pre-infection CD4 levels nor the exact timing of the initial infection were known, as patients were deemed to be in acute stage of infection based on viremia in the face of negative serology. In contrast, studies of SIV-infected macaques were based on restoration to pre-inoculation CD4 levels. Furthermore, therapy in the macaques was initiated at six weeks of infection [7], which is likely much earlier than the human patients in the current study. Second, the current study examined rectum/colon CD4^+^ T cells, whereas macaque studies are usually performed using samples from the jejunum. The rectum/colon is a mix of immune inductive (organized lymphoid tissues) and effector sites (diffuse lamina propria) whereas the jejunum contains almost no immune inductive sites. This is reflected in the lymphoid composition of each tissue: the jejunum contains mostly memory CD4^+^ T cells, while the colon contains a larger proportion of naïve CD4^+^ T cells. This makes it difficult to extrapolate comparisons of T cell subsets from these disparate sites. Third, the percentages of “activated” T cell subsets remaining after treatment need to be interpreted with caution, as these are “snapshot” data observed during what is now believed to be a very dynamic process. For example, detection of increased percentages of “activated” CD4^+^ T cells in a tissue that has undergone massive CD4^+^ T cell depletion could be interpreted either as an immune response to the infection or as a selective loss of “resting” CD4^+^ T cells. However, given that the study also found that higher activation levels were associated with poorer mucosal reconstitution, the more logical interpretation is that HIV infection results in continual activation, turnover, and destruction of intestinal CD4^+^ T cells. Finally, the low level of infected cells detected in the intestine of patients on ART should be interpreted with caution, as the conclusion was based on small biopsy samples taken from the largest immune organ in the body. Detecting even a small number of infected cells in these pinch biopsies could reflect a very large number of infected cells throughout the intestine.

## Conclusions and Clinical Implications

In conclusion, the findings reported by Mehandru et al. indicate that most patients who initiate therapy as early as possible after HIV infection still do not experience complete restoration of intestinal CD4^+^ T cells to baseline levels, regardless of the duration of therapy. Instead, HIV infection results in a continuous state of activation in the intestinal immune system that is not reflected in peripheral lymphoid tissues. Combined, the data provide evidence that intestinal inflammation and continual infection, destruction, and turnover of CD4^+^ T cells occur in patients on ART, suggesting that the major battle against HIV occurs in sequestered mucosal tissue sites. This important observation suggests a number of potential therapeutic strategies for further research; for example, the investigation of drugs with better intestinal tissue distribution and perhaps the exploration of mechanisms to reduce immune activation in mucosal tissues to more effectively combat HIV infection.

## Abbreviations

ART: antiretroviral therapy
SIV: simian immunodeficiency virus
